# Exploring the Antiparasitic Activity of *Tris*-1,3,4-Thiadiazoles against *Toxoplasma gondii*-Infected Mice

**DOI:** 10.3390/molecules27072246

**Published:** 2022-03-30

**Authors:** Tahani M. Almutairi, Nadjet Rezki, Mohamed Reda Aouad, Mohamed Hagar, Basant A. Bakr, Moaaz T. Hamed, Maha Khairy Hassen, Bassma H. Elwakil, Esraa Abdelhamid Moneer

**Affiliations:** 1 Department of Chemistry, College of Science, King Saud University, P.O. Box 2455, Riyadh 11451, Saudi Arabia; talmutari1@ksu.edu.sa; 2 Department of Chemistry, Faculty of Science, Taibah University, Al-Madinah Al-Munawarah 30002, Saudi Arabia; nadjetrezki@yahoo.fr (N.R.); aouadmohamedreda@yahoo.fr (M.R.A.); 3 Department of Chemistry, Faculty of Science, Alexandria University, Alexandria 21321, Egypt; 4 Department of Zoology, Faculty of Science, Alexandria University, Alexandria 21321, Egypt; bassant.kamal@pua.edu.eg; 5 Department of Botany and Microbiology, Faculty of Science, Alexandria University, Alexandria 21321, Egypt; moazt258@gmail.com; 6 Department of Medical Laboratory Technology, Faculty of Applied Health Sciences Technology, Pharos University in Alexandria, Alexandria 21321, Egypt; maha.khairy@pua.edu.eg (M.K.H.); esraa.moneer@pua.edu.eg (E.A.M.)

**Keywords:** *tris-*1,3,4-thiadiazoles, antiparasite, *Toxoplasma gondii*, in vivo study, immunological studies, docking studies

## Abstract

Nitrogen-containing atoms in their core structures have been exclusive building blocks in drug discovery and development. One of the most significant and well-known heterocycles is the 1,3,4-thidiazole nucleus, which is found in a wide range of natural products and therapeutic agents. In the present work, certain *tris*-1,3,4-thiadiazole derivatives (**6**, **7**) were synthesized through a multi-step synthesis approach. All synthesized compounds were characterized using different spectroscopic tools. Previously, thiadiazole compounds as anti-*Toxoplasma gondii* agents have been conducted and reported in vitro. However, this is the first study to test the anti-*Toxoplasma gondii* activity of manufactured molecular hybrids thiadiazole in an infected mouse model with the acute RH strain of *T. gondii*. All the observed results demonstrated compound (**7**)’s powerful activity, with a considerable reduction in the parasite count reaching 82.6% in brain tissues, followed by liver and spleen tissues (65.35 and 64.81%, respectively). Inflammatory and anti-inflammatory cytokines assessments proved that Compound 7 possesses potent antiparasitic effect. Furthermore, docking tests against *Tg*CDPK1 and ROP18 kinase (two major enzymes involved in parasite invasion and egression) demonstrated compound **7**’s higher potency compared to compound **6** and megazol. According to the mentioned results, *tris*-1,3,4-thiadiazole derivatives under test can be employed as potent antiparasitic agents against the acute RH strain of *T. gondii*.

## 1. Introduction

**Figure 1:**
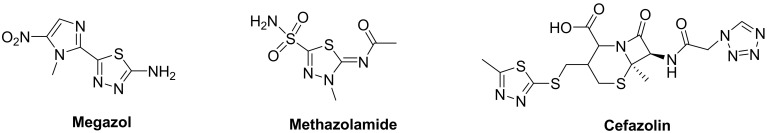
Commercially available thiadiazole drugs.

*Toxoplasma gondii* is an obligate intracellular parasite that can spread to all warm-blooded vertebrates, including humans, causing a disease called toxoplasmosis [1,2]. The seropositive rates ranged from 10 to over 90% [1,2,3]. One third of the world’s population is suffering from toxoplasmosis [3]. The T. gondii infection chain ideally begins with the ingestion of oocysts (from a cat’s faeces or undercooked meat), followed by the release of sporozoites and bradyzoites from the ingested oocysts to invade intestinal cells, where they are converted into tachyzoites. The tachyzoites then spread to other organs through the blood or lymphatic system. Consequently, tachyzoites can cause an acute infection (AI) or a chronic infection (CI) [4]. Immune responses differ during acute (proliferative), chronic, and/or latent (dormant) stages based on differences in virulence, phenotype, and clonal lineages of the strain’s clinical consequences [4,5]. Serum Th1 cytokines (e.g., interleukin (IL)-12, IL-18, interferon (IFN)- and tumour necrosis factor (TNF)) rise during the acute infection stage of the highly virulent RH strain, followed by mouse mortality 8 to 10 days post-infection [6]. For example, IL-12, TNF-, and IFN- proved to inhibit parasitic growth (both in vivo and in vitro), whereas TGF- and IL-10 regulate the excessive inflammatory response in the brain [7]. The ideal toxoplasmosis treatment regimen relies on the inhibition of the parasitic invasion and egression in the host cells, which will lead to inhibiting the parasitic replication (since *T. gondii* is an obligate intracellular parasite). Two main enzymes are responsible for parasitic invasion into the host cells, calcium-dependent protein kinase 1 (*Tg*CDPK1) and ROP18 kinase (*Tg*ROP18) [8] (members of the serine/threonine protein kinase family) [9]. Many potent compounds have been investigated as kinase inhibitors and were called “bumped kinase inhibitors” (BKIs) [10]. Many medications were used to treat toxoplasmosis, including pyrimethamine, spiramycin, trimethoprim, sulfadiazine, sulfamethoxazole, and sulfadoxine, but 80% of patients relapsed, while others suffered from bone marrow suppression, intolerance or allergic reactions, and the drugs’ poor cell membrane crossing [11]. Researchers’ attention was directed to mesoionic systems with highly polarizable characteristics to enhance the anti-toxoplasmosis targets, with distinctive affinities and cellular membrane crossing enhancements. The most important mesoionic systems were the heterocycles containing both nitrogen and sulphur atoms, such as thiadiazoles, which are potent biologically active compounds. Among the four thiadiazole isomeric forms (e.g., 1,2,3-thiadiazole, 1,2,4-thiadiazole, 1,2,5-thiadiazole, and 1,3,4-thiadiazole), the 1,3,4-thiadiazole ring is the most prevalent in medically significant synthetic compounds [11,12,13]. The 1,3,4-thidiazole nucleus is present as a core structural component in an array of drug categories, such as antifungal [14], antimicrobial [15], anti-inflammatory [16], anticancer [17,18], antiparasitic [19], and antiviral [20] agents. Molecular hybridization, on the other hand, is a new area in drug development that has the potential to lessen side effects and avoid drug resistance. In comparison with their single equivalents, integrating two or more pharmacophoric cores in a single molecular framework resulted in novel hybrid drugs with improved selectivity, profile, efficacy, and affinity [21,22,23]. Furthermore, thiadiazole drugs, such as cefazolin (first generation cephalosporin) and sulfamethizole as antimicrobial sulfonamides, acetazolamide, methazolamide, and carbonic anhydrase inhibitors as diuretic drugs, and megazol as an antiparasitic drug, are widely produced and distributed in the global market [24] (Figure 1). Megazol was firstly synthesized as an antimicrobial agent and then proved to be a powerful antiprotozoal agent [24].

**Figure 2:**
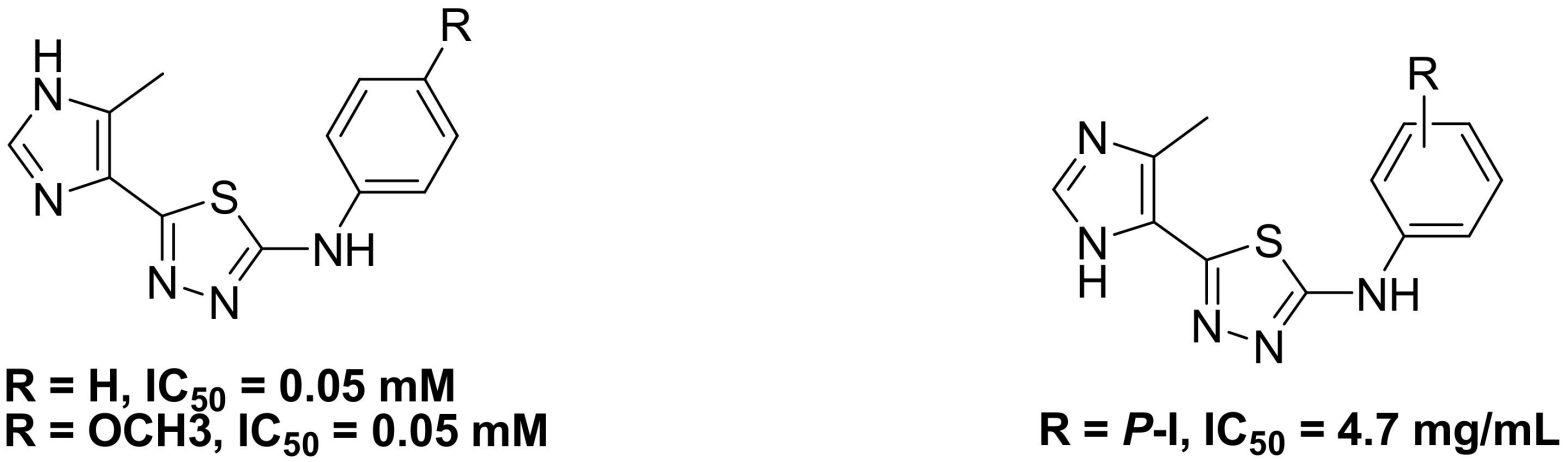
Structure-based rational design of the synthesized 1,3,4-thidiazoles.

Liesen et al. [19] synthesized a series of 1,3,4-thiadiazoles as anti-*Toxoplasma gondii* agents. The compounds showed a potent antiparasitic activity at 0.1 mM in vitro, and IC_50_ reached 0.05 mM. The selectivity of the prepared compounds against the intracellular parasite *T. gondii* was investigated using structure–activity relationships, which demonstrated that the electronic nature of the attached substituents on the phenyl ring had no effect on anti-*T. gondii* activity (Figure 2).

Węglińska et al. [11] synthesized 1,3,4-thiadiazoles that could effectively inhibit the proliferation of *Toxoplasma gondii* in vitro with an IC_50_ of 4.70 µg/mL, which were considered early hit compounds for the future design of anti-*Toxoplasma* agents that effectively inhibited the parasite invasion and intracellular proliferation via direct action on both tachyzoites and parasitophorous vacuoles (Figure 2).

In view of the foregoing concerns, in the present work, we anticipated the molecular binding of the synthesized disubstituted 1,3,4-thidiazole unit with two cores of 1,3,4-thiadiazole in one framework through a flexible propyl linker, in order to investigate their synergistic effects. The chemical structure of the designed molecular hybrids could provide enhanced interactions against *Toxoplasma gondii*-infected mice.

## 2. Results

### 2.1. Chemistry

#### 2.1.1. Synthesis of the Targeted Compounds

**Scheme 1:**
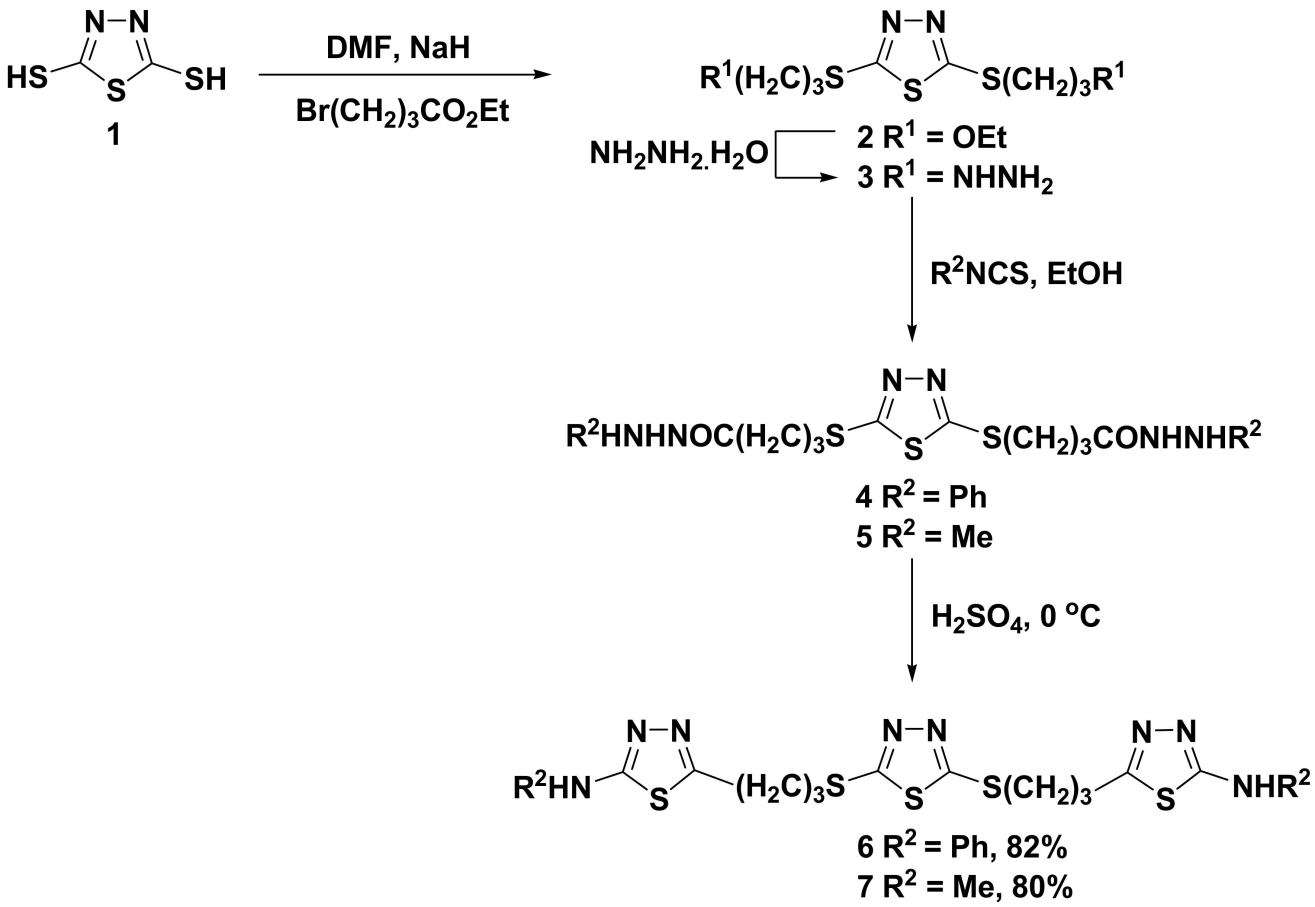
Synthetic routes for the synthesis of *tris*-1,3,4-thiadiazoles **6**–**7**.

The aim *tris*-thiadiazoles **6** and **7** were synthesized through a multi-step synthesis approach that was initiated with 2,5-dimercapto-1,3,4-thiadiazole (**1**) [25] based on the previously stated procedure [17] (Scheme 1). Thus, the basic alkylation of the focused 2,5-dimercapto-1,3,4-thiadiazole (**1**) with 4-ethylbromobutyrate in dimethylformamide (DMF) as solvent and sodium hydride as basic catalyst resulted in the formation of the corresponding 1,3,4-thiadiazole tethering the thiobutanoate side chain as ester functionality **2**, which underwent hydrazinolysis via its thermal treatment with hydrazine hydrate for 6 h, which gave the respected acid hydrazide **3** (Scheme 1).

The condensation of the resulting hydrazide **3** with phenyl/methyl isothiocyanates resulted in good yields (86–89%) of the key acid thiosemicarbazide-based-1,3,4-thiadiazoles **4** and **5**. The targeted 2-aminophenyl/methyl-1,3,4-thiadiazoles **6** and **7** were successfully synthesized through the action of sulfuric acid (H_2_SO_4_) on the latest precursors **4** and **5** at 0 °C.

#### 2.1.2. Spectroscopic Characterization of the Targeted Compounds

Different spectroscopic experiments were used to elucidate the structures of the resultant *tris*-1,3,4-thiadiazoles **6** and **7**, as well as their intermediates, such as IR, ^1^H, ^13^C NMR, MS, and elemental analyses. The alkylation reaction was supported by the disappearance of the characteristic absorption band of the thiol groups and the appearance of new diagnostic absorption bands between 2879 and 2936 cm^−1^ belonging to the aliphatic (C-H) stretching vibrations of the ester side chain. The C=O and C-O groups of esters 2 were also observed as distinct bands at 1740 and 1270 cm^−1^, respectively. The investigation of the ^1^H NMR spectrum clearly revealed the resonance of the ester protons (CH_2_CH_3_) at triplets and quartets at δ_H_ 2.06 ppm and δ_H_ 4.08–4.12 ppm, respectively. The butyric methylene protons (CH_2_CH_2_CH_2_, CH_2_CO and SCH_2_) were recorded as two multiplets at δ_H_ 1.78–1.82 and 1.87–1.93 ppm, and a triplet at δ_H_ 3.32 ppm, respectively. On the other hand, the ^13^C-NMR spectrum of compound **2** was inconsistent with its framed pattern. Thus, a new signal appeared at δ_C_ 20.94 ppm assigned to the methyl carbons, while the methylene carbons were recorded at δ_C_ 25.88, 27.61, 33.66, and 63.63 ppm. The signals assigned to C=N and C=O carbons were observed at δ_C_ 164.86 and 170.99 ppm, respectively.

The success of the hydrazinolysis reaction of the bis-ester **2** was supported by the spectral data of the resultant acid hydrazide **3**. Its IR clearly showed the presence of the characteristic NH and NH_2_ groups of the hydrazide linkage at 3250–3400 cm^−1^. The ^1^H NMR spectrum also approved the presence of such a linkage by the presence of two singlets resonating at δ_H_ 4.22 and 9.02 ppm assigned to the NH_2_ and NH protons, respectively. All remaining protons were recorded in their appropriate chemical shifts (see Experimental Section). The carbonyl amide (NHC=O) carbons appeared as significant signals at δ_C_ 166.82 ppm, giving conclusive proof for the formation of the bis-acid hydrazide **3**.

The structures of bis-acid thiosemicarbazides **4** and **5** were deduced based on their spectral results. Their IR spectra exhibited the presence of a new absorption band at 1280–1310 cm^−1^ belonging to the thiocarbonyl group (C=S). The absence of the hydrazide (NH_2_, NH) protons and the presence of distinctive NH thiosemicarbazide in the area of δ_H_ 7.88–9.67 ppm in their ^1^H-NMR spectra supported the formation of the precursors, bis-acid thiosemicarbazides **4**–**5**. In addition, new signals were recorded in the aromatic and aliphatic regions belonging to the phenyl and methyl protons (see Experimental Section). Moreover, the diagnostic thione carbons (C=S) were observed at δ_C_ 173.44–174.90 ppm in their ^13^C-NMR spectra. The rest of the carbons were recorded in their respective areas.

The spectral data of the thiadiazoles **6**–**7** disclosed the disappearance of the carbonyl (C=O) and thiocarbonyl groups in their IR spectra and the appearance of distinctive absorption peaks near 1624–1657 cm^−1^ related to the C=N group. Furthermore, all the chemical shifts observed in their ^1^H-NMR and ^13^C-NMR spectra were all justified by the suggested structures **6**–**7**. Their ^1^H-NMR spectra revealed the absence of signals associated with the -CONH and -NHCSNH- of their respective thiosemicarbazides **4**–**5**. The characteristic exocyclic NH proton at position 2 of the 1,3,4-thiadiazole core resonated at δ_H_ 9.87–10.13 ppm. The phenyl and methyl protons were recorded in their appropriate areas. The signals belonging to the same methyl and phenyl carbons were observed at δ_C_ 38.69 and 116.06–158.09 ppm in the ^13^C-NMR spectra. The spectra also revealed the absence of the signals related to the carbonyl (C=O) and thiocarbonyl (C=S) groups of their precursors **4** and **5**.

### 2.2. Acute Toxicity

**Figure 3:**
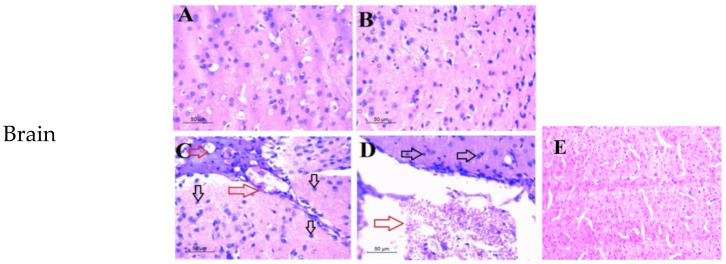

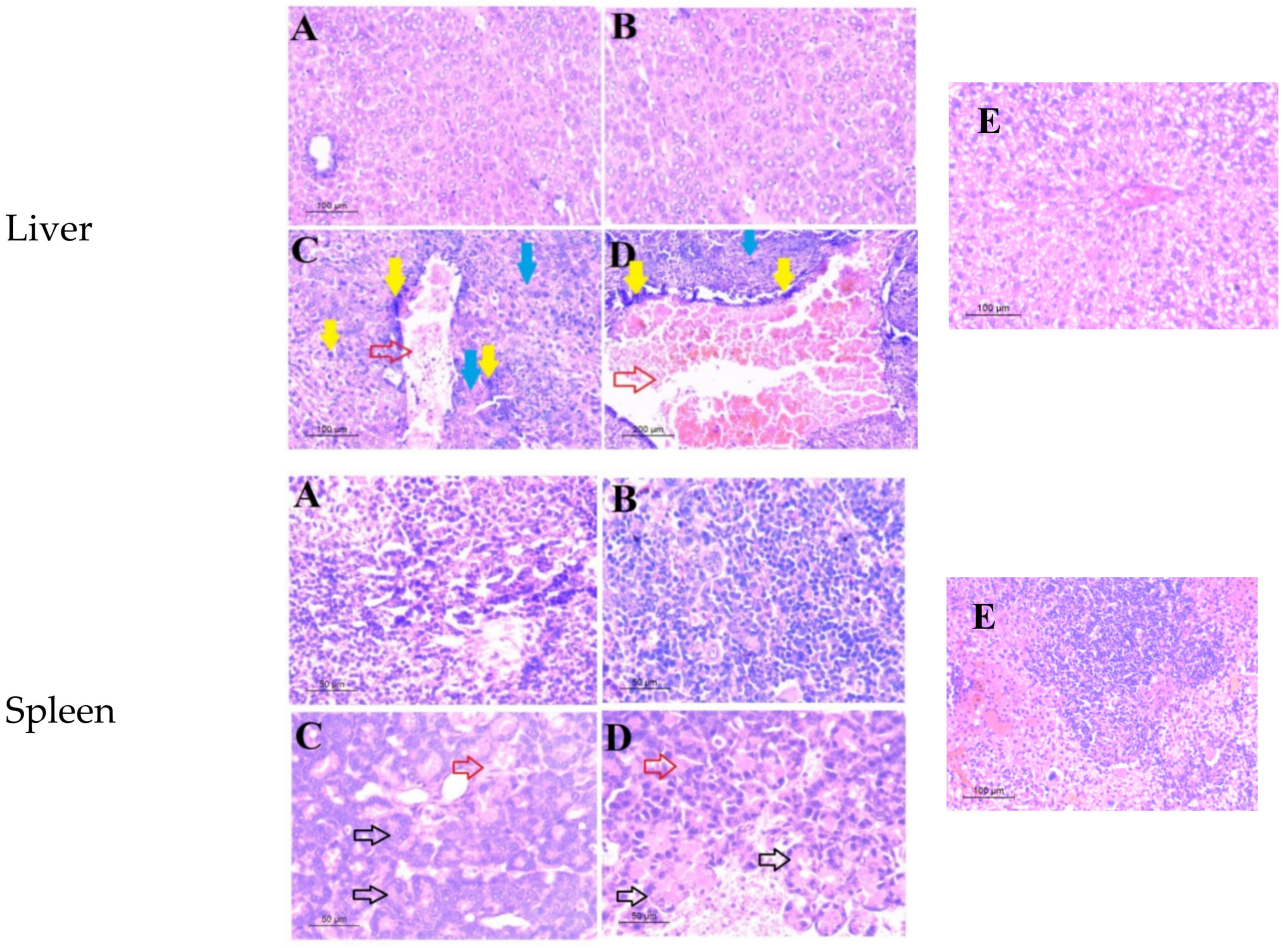
Photomicrograph of three collected organs (brain, liver, spleen) showing the toxicity effect of compounds **6**,**7** at histopathological level, in which (**A**,**B**) represent the low dose of compounds **6**,**7**, respectively, showing normal histological architecture with reference to the control one (**E**). (**C**,**D**) represent the high dose of compound **6**,**7**, respectively, showing in brain sections congested cerebral blood vessels (red arrows) and the necrosis of neurons (black arrows). Liver sectors (**C**,**D**) show cellular toxicity with dilated, congested blood vessels (red arrows), necrosis (yellow arrows), and blood sinusoid congestion (blue arrows). Spleen sectors (**C**,**D**) show red pulp hyperaemia (red arrows) and Langham’s cells (black arrows).

For both drugs (**6** and **7**), H&E-stained mouse brain slides demonstrated normal cerebral cortex histology in the low-dosage-treated groups. Meanwhile, high dosages of both drugs caused neuronal necrosis in the cerebral cortex. In addition, both groups had vascular congestion, especially focal bleeding in the high dosage of the compound **7** treated group. Furthermore, dilated and blocked blood vessels, as well as blood sinusoids, were observed in the high-dose-treated groups’ liver sections as signs of toxicity. Moreover, the low-dose-treated groups showed normal white pulp, red pulp, and spleen trabecularity, but the high-dose-treated groups showed red pulp hyperaemia (Figure 3).

### 2.3. Clinical Study

No signs were noticed in the uninfected mice in group I. Meanwhile, in group II mice (infected but did not receive any treatment), some observations were recorded, e.g., the mice had hair erections and their mobility and feeding/drinking activities were decreased. However, groups III, IV, and V (infected treated mice) appeared relatively healthy with normal food intake.

### 2.4. Parasitological Study

#### 2.4.1. Parasite Count and Percent Reduction (%R)

**Figure 4:**
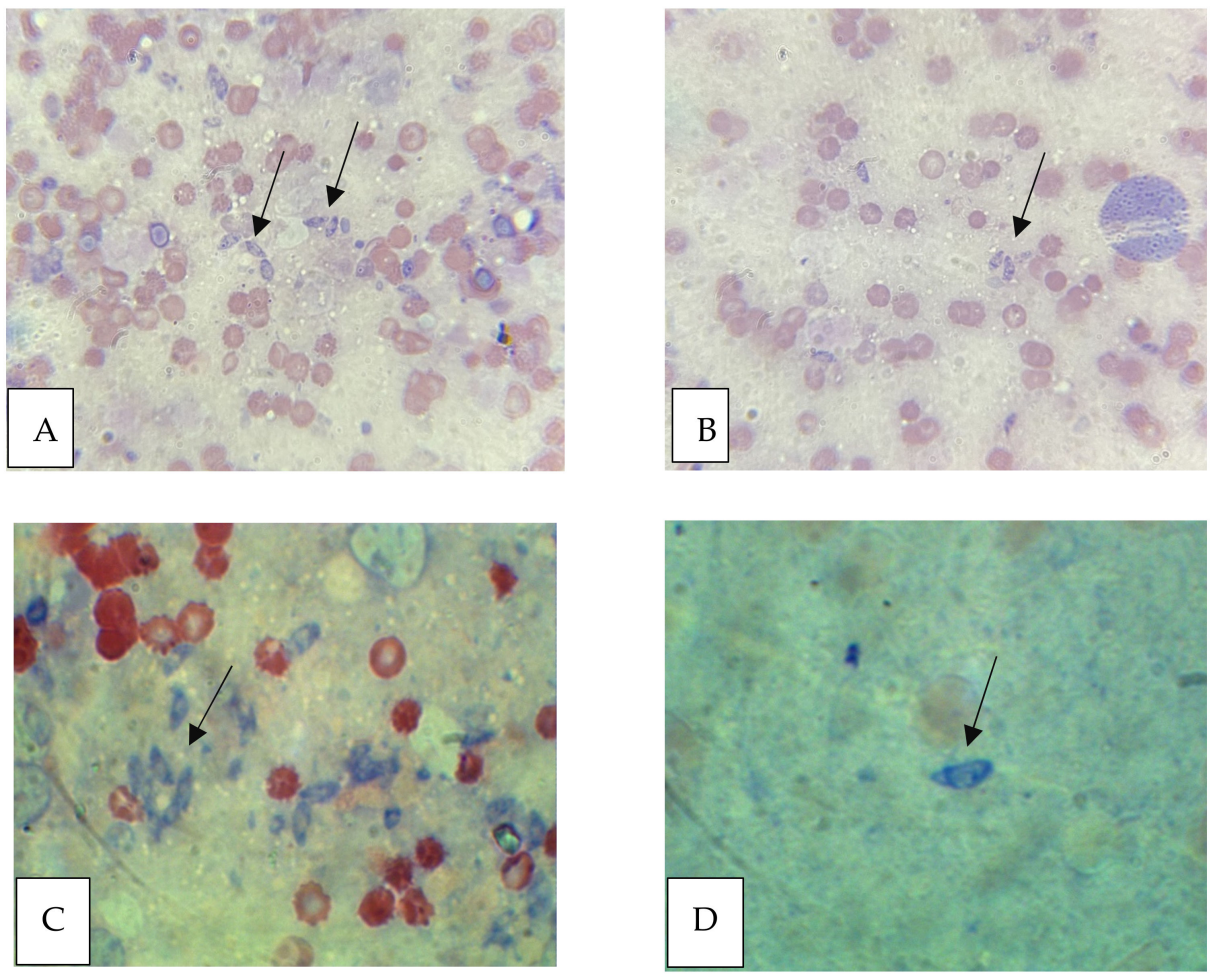

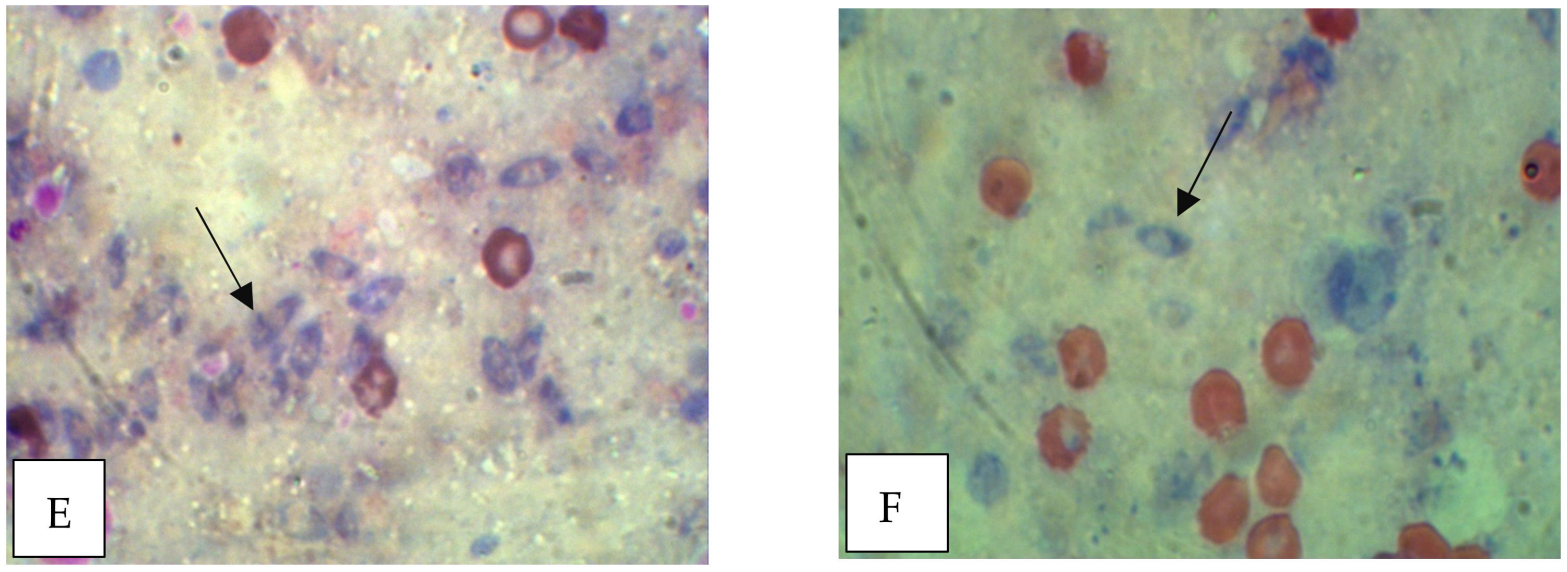
*T. gondii* tachyzoites in Giemsa-stained liver impression smear of infected untreated control (**A**) and infected treated mice (**B**), ×1000. *T. gondii* tachyzoites in Giemsa-stained spleen impression smears of infected untreated control (**C**) and in infected treated mice (**D**), ×1000. *T. gondii* tachyzoites in brain impression smear of infected untreated control (**E**) and in infected treated mice brain impression smear (**F**), ×1000.

In the present work, 1,3,4-thiadiazole derivatives, namely **6** and **7**, were evaluated in addition to the commercially available thiadiazole antiparasitic drug (megazol) for the treatment of mice infected with the *T. gondii* virulent RH strain. The mean tachyzoite counts in the infected untreated group were 10.1, 5.4, and 2.3 in the liver, spleen, and brain, respectively. Regarding treated mice, the lowest mean tachyzoite count and the highest percentage reduction were detected in mice that received compound **7** (Figure 4). There was a significant reduction in parasite count in the infected group treated with compound **6** and the infected group treated with compound **7**, as compared to the infected untreated group and the infected, treated megazol mice group, as shown in Table 1. These results were inconsistent with other studies which declared the powerful effect of 1,3,4-thiadiazole by causing a significant decrease in the infection percentage, as well as the mean number of tachyzoites [11,19]. Węglińska et al. [11] investigated the 1,3,4-thiadiazoles isomers and the corresponding anti-*T. gondii* activity, and it was reported that the *meta*-isomer showed more activity than its *para* counterpart (*meta* > *para* > *ortho*), indicating a significant relationship between the substitution pattern and the anti-*T. gondii* activity of the synthesized 1,3,4-thiadiazole. Other reported findings were that anti-*T. gondii* action by the 1,3,4-thiadiazole was not only specific but also did not result in any host cells toxicity.

#### 2.4.2. Morphological Study of *T. gondii* Tachyzoites

**Figure 5:**
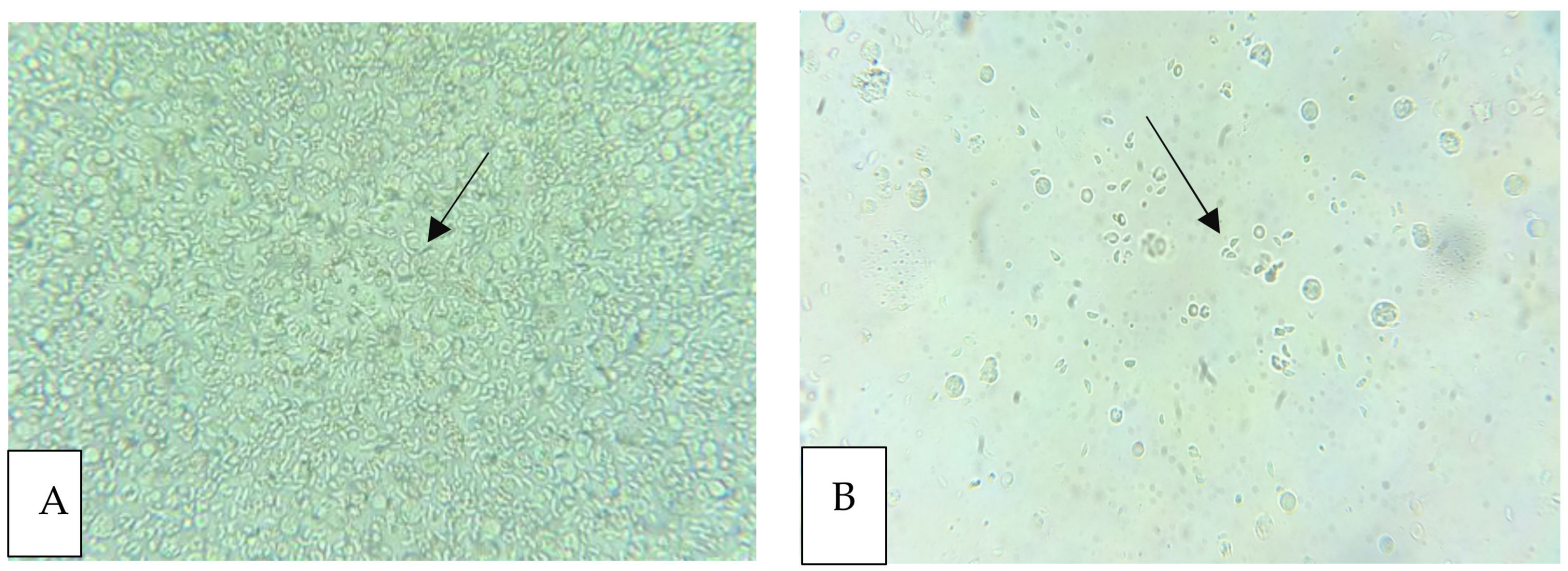
Light microscopic examination of the tachyzoites in the peritoneal exudates of RH strain-infected mice. (**A**). Infected control group showing the tachyzoites with a normal movement and shape (**B**). Infected group treated with showing tachyzoites with sluggish movement and deformed crescent shape.

**Figure 6:**
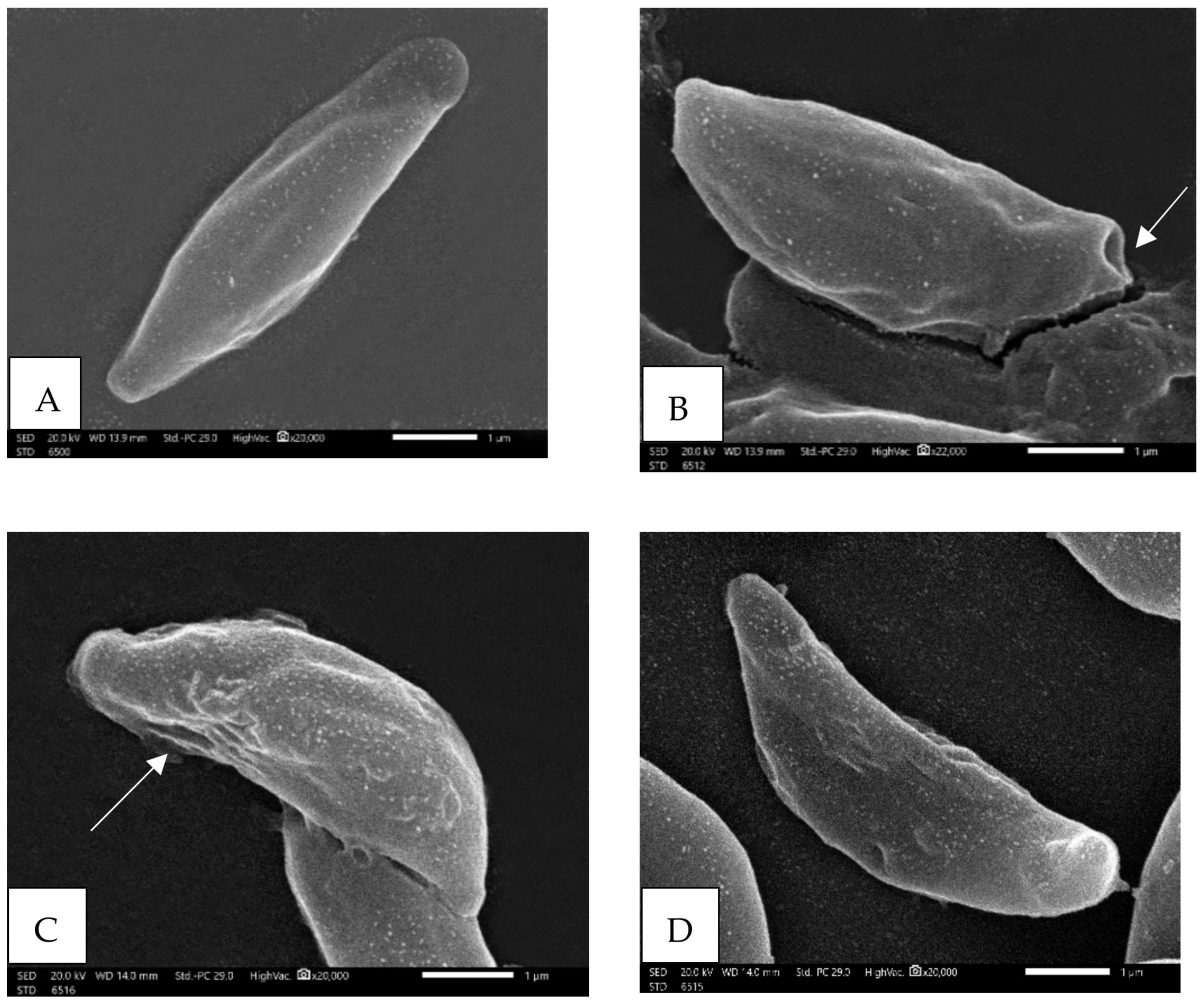
*Toxoplasma* gondii tachyzoite in the studied groups by SEM. (**A**) An SEM of *Toxoplasma gondii* tachyzoite in infected untreated group showing completely regular smooth surface and evident conoid (×20,000). (**B**) A SEM of *Toxoplasma gondii* tachyzoite in infected group treated with compound **6** showing irregular surface and a hole in the surface (arrow) (×22,000). (**C**) An SEM of *Toxoplasma gondii* tachyzoite in infected group treated with compound **7** showing multiple ridges and irregular papules and dimples (arrow) and disorganized conoid (×20,000). (**D**) An SEM of *Toxoplasma gondii* tachyzoite in infected group received megazol showing irregular surface and multiple ridges (×20,000).

In the infected untreated control group, light microscopic examination of the tachyzoites in the peritoneal exudates revealed normal tachyzoites movement and form. Tachyzoites in both groups III and IV (treated with compound **6** and compound **7**) showed sluggish movement with a deformed crescent shape (Figure 5). As demonstrated by SEM, the untreated parasite control group showed normal smooth surfaces of *T. gondii* tachyzoites. On the other hand, tachyzoites from treated groups (III, IV and V) showed distortion and deformation in their shapes (Figure 6). Previous in vitro studies against intracellular *T. gondii* confirmed our observed results with the complete morphological disorganization of tachyzoites after 24 h of incubation with the thiadiazole derivatives [11].

### 2.5. Inflammatory and Anti-Inflammatory Cytokines in Infected and Treated Groups

The inflammatory mechanisms as a response to *T. gondii* infection were investigated through some inflammatory and anti-inflammatory cytokine (TNF-ɑ, IL-10, IL-6, and IL-1B) assessments. The data in Table 2 revealed that the inflammatory and anti-inflammatory cytokine concentrations were significantly high in the infected non-treated mice (almost double the normal concentration), as an indication of the progression of the acute infection (AI) stage. It is worth noting that the mice group IV treated with compound **7** showed a lower inflammatory response, and the cytokine concentrations were significantly close to those of the healthy non-infected mice. *T. gondii* infection causes a variety of immune responses, including inflammatory and anti-inflammatory cytokine expression increments, as well as microglial cell activation and proliferation [7]. Hence, compound **7** may be a new powerful anti-toxoplasmosis agent with an immune modulatory effect.

### 2.6. Histopathological Study

#### 2.6.1. Liver

The control group’s H&E-stained liver sections exhibited normal hepatic architecture; normal hepatic lobules seemed to be composed of intact hepatocytes grouped into cords that extended from the central vein. They were polyhedral in form, with spherical vesicular nuclei in the centre. The hepatic sinusoids were observed as thin cavities between the hepatic cords. A normal portal triad was also detected. This triad consisted of a portal vein with a big lumen and a thin wall, a hepatic artery with a small lumen and a thick wall containing smooth muscle fibres, and one bile duct, all of which were surrounded by a modest amount of connective tissue. Negative control mice livers were examined histopathologically and indicated a number of cellular and lobular abnormalities. Some hepatocytes had inflated cytoplasm with nuclear alterations, while others had hazy vacuolated cytoplasm and ambiguous cell borders, indicating early symptoms of apoptosis. Nuclear division, nuclear eccentricity, pyknosis, and necrosis are examples of nuclear alterations. The dilated blood vessel was clogged. The central vein was dilated, congested, and infiltrated with mononuclear cells. The majority of the blood sinusoids were dilated and congested.

**Figure 7:**
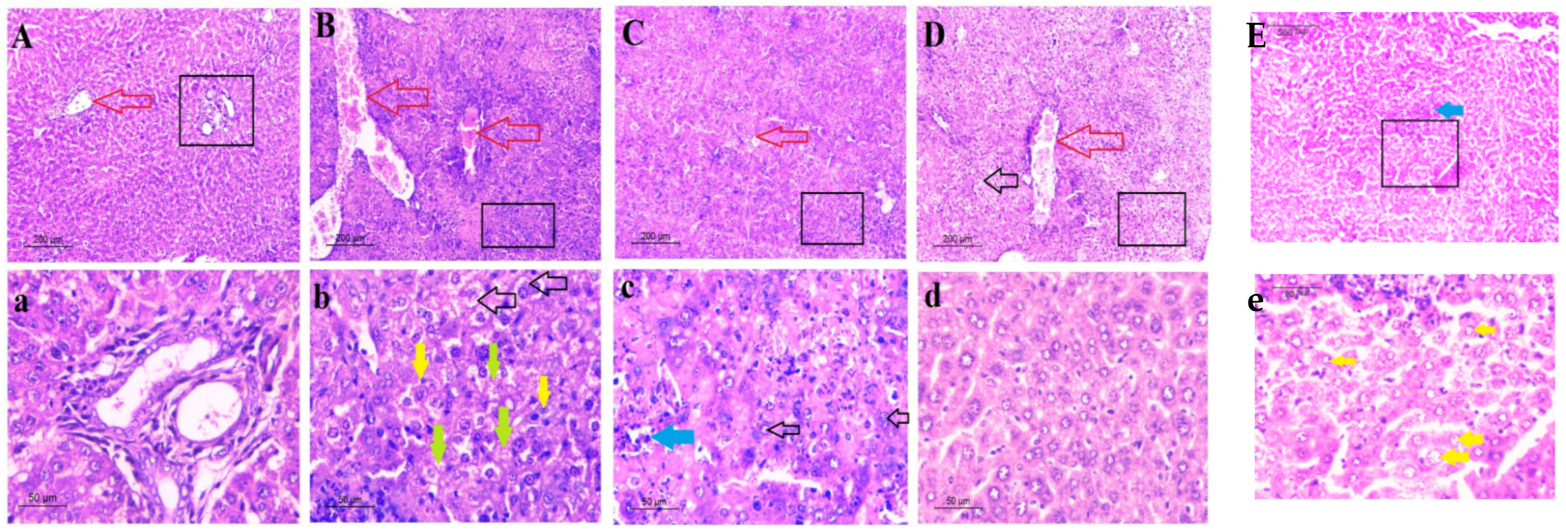
Photomicrograph of liver sections of male mice in which (**A**) control group (group I) showing normal cytoarchitecture with normal portal traid ((**a**), 1000×, H&E stain)) and central vein (red arrow); (**B**) group II showing numerous blood vessels dilated and congested (red arrows); ((**b**), 1000×, H&E stain)) ballooned hepatocytes (black arrows); pyknotic nuclei (yellow arrows); congested blood sinusoids (green arrows); (**C**) treated group III hepatocytes have eccentric nuclei (black arrows); necrosis (blue arrow); (**D**) treated group IV mild affection of the hepatic lobule with semi normal arrangement of hepatocytes (black arrow); dilated central vein (red arrow); and (**E**) treated group V showed pyknotic nuclei (yellow arrow) and necrosis (blue arrows) (400×, H&E stain) ((**a**–**e**) represented the higher magnification (1000×, H&E stain).

The hepatic architecture was more or less intact in the compound **6** treated group. There were no visible symptoms of congestion or dilatation in the central vein. Hepatocytes were arranged irregularly around central veins with spherical vesicles, and necrosis was still visible. On the other hand, the mice group IV histopathological investigations showed that there was still some dilation of the central vein (Figure 7). Cellular invasion is almost extinct. There were no necrotic foci found. Congestion in the blood sinusoids was not observed. On the other hand, Group V demonstrated a temporary period between the two treatment groups, with normal hepatocyte appearance with some necrotic appearance and degenerated hepatic cells with pyknotic nuclei.

#### 2.6.2. Spleen

**Figure 8:**
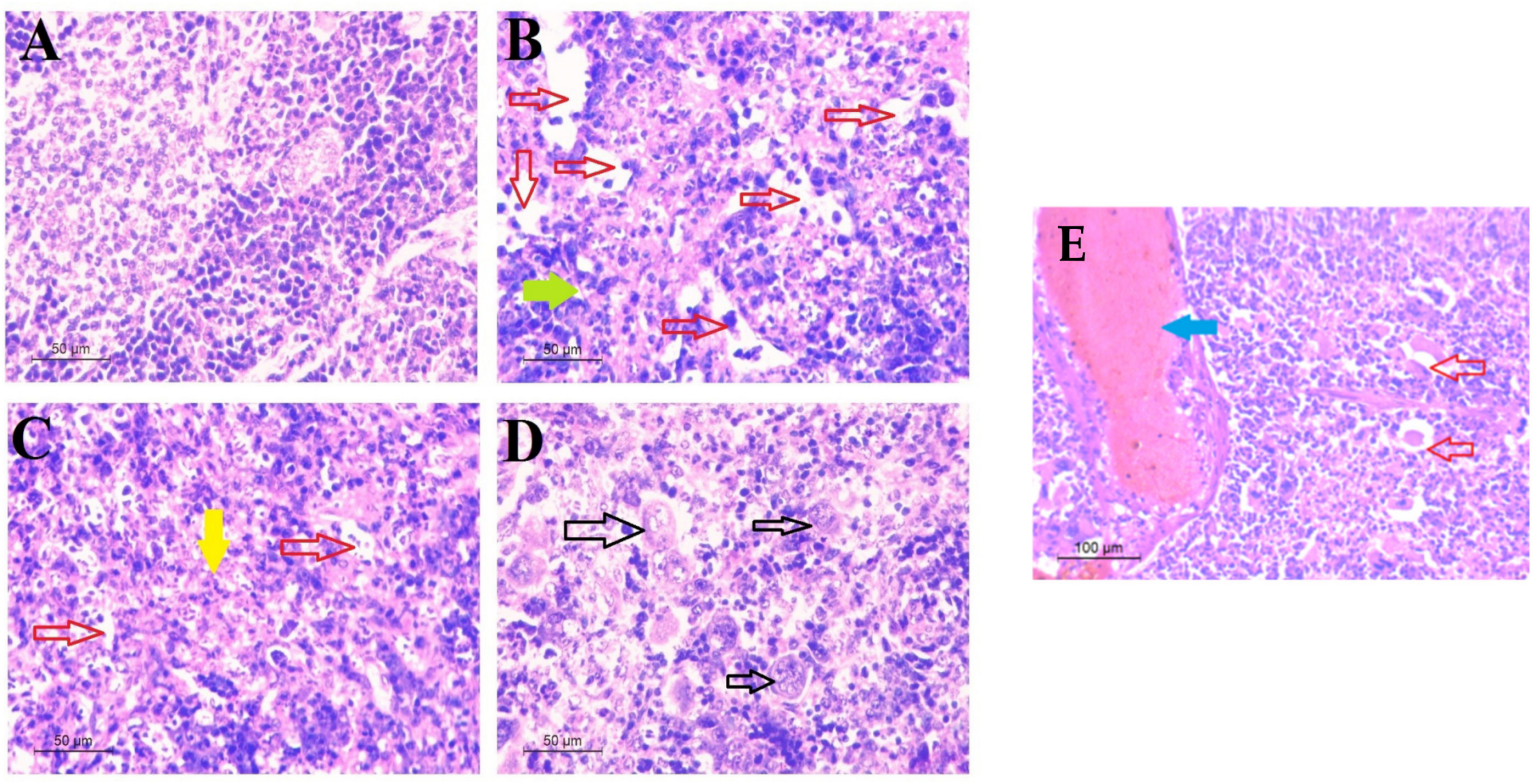
Microscopic sections from spleen (**A**) control mice control group showed clearly delineated lymphocytes, lymphnode and red pulp, (**B**) *T. gondii*-infected control mouse (group II) showing degeneration (red arrows) and fibrosis (green arrow), (**C**) *T. gondii*-infected mouse treated with compound **6** (group III) showing degeneration (red arrows) and hematopoiesis (yellow arrow); (**D**) *T. gondii* mouse treated with compound **7** (group IV) showed a greater number of lymphoid follicles (black arrows) and (**E**) *T. gondii* mouse treated with megazol, where blue arrow refers to dilated blood vessel and red arrow refers to tissue degeneration (400×, H&E stain).

The control group’s splenic architecture was described as well delineated white and red pulp with contiguous trabecular all throughout the tissues. In the white pulp, the usual patterns of periarteriolar lymphoid sheaths (PALS) and lymphoid follicles were also visible. The most striking discovery was substantial T cell depletion in the PALS in the non-treated mice spleen, which showed significant degeneration, necrosis, and fibrosis. There were also alterations in the size and form of the spleen’s white pulp as described by Gaafar et al. [26]. In group III, further results included infrequent clusters of plasma cells in the PALS and enhanced erythroid hematopoiesis in the red pulp (Figure 8). Concerning group IV, the histological features were similar to those of control group I, with a greater number of lymphoid follicles compared to treated group III. Group V showed almost normal histological appearance, with minor degeneration and large dilated blood vessels, indicating that this therapy had an angiogenesis impact.

#### 2.6.3. Brain

**Figure 9:**
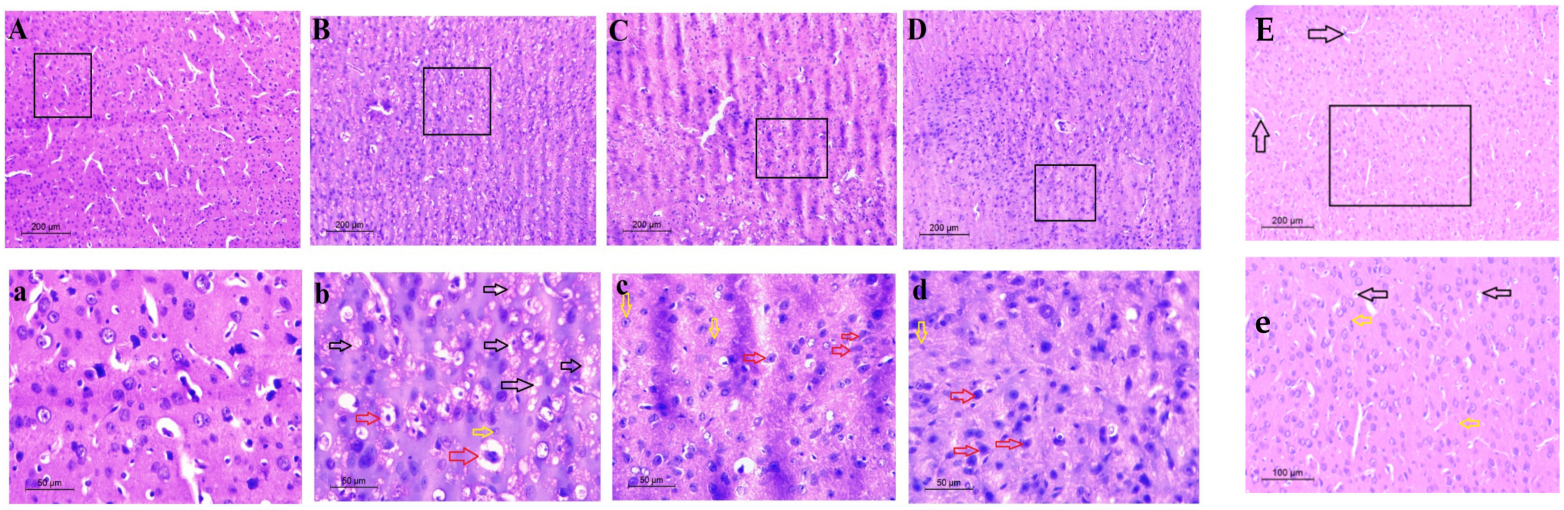
Photomicrograph of mice cerebral cortex. (**A**) Group (I) showed normal neurons with widespread glial cells in between, (**B**) Group II showed dystrophic alterations in the form of shrunken hyperchromatic, irregular with chromatolysis (red arrows) and aberrant Nissl granule (yellow arrows), with numerous degenerate and vacuolated neurocytes (black arrows). (**C**–**E**) Treated groups (Groups III, IV and V) respectively show more or less normal neurons (Red arrows) with glial cells (yellow arrows) (400×, H&E stain) ((**a**–**e**) represented the higher magnification (1000×, H&E stain)).

Brain samples of experimental mice were examined by H&E stain. The typical architecture and cell density features of healthy tissues were not altered in any of the control brain tissues (group I). Tissue sections revealed pink-coloured cells with purple-coloured cytoplasm and nuclei, with no visible alterations in the chromatin organization. On the other hand, Group II sections showed unevenly organized cells with larger densities at seemingly less wounded locations, lower cell density, and a considerable increase in the vacuolated neurocytes, as reported by Fuentes-Castro et al. [27]. According to Parlog et al. [28], inflammatory infiltration, gliosis, and the existence of *T. gondii* cysts were seen in the brains; these are the key pathological features of the chronic infection phase. Significantly more or fewer normal neurons and glial nodules were seen in the treated groups. No parasite markers were found in any of the investigated sections. Interestingly, group IV has more prominent pyramidal neurons with basophilic cytoplasm and pale nuclei, as well as glial growth throughout the brain (Figure 9).

In comparison to the two treated groups, Group V had the most significant histopathological findings. Although some neuronal vacuolations remain, their low quantity and the normal appearance of neurons reflect the drug’s anti-tachyzoite activity (Figure 9).

### 2.7. Molecular Docking

**Figure 10:**
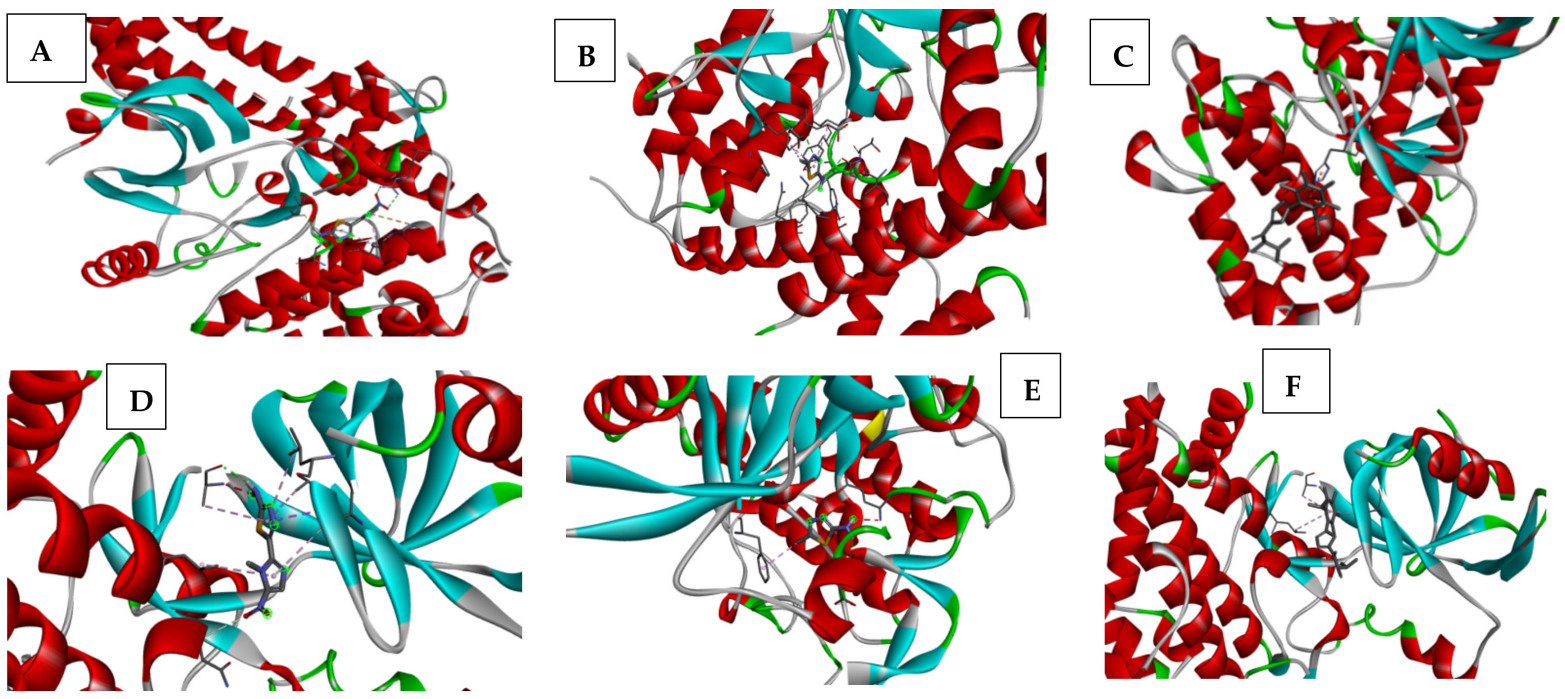
Predicted binding modes of the standard drug megazol, compound **6** and **7** against *Tg*CDPK1 ((**A**–**C**) respectively) and *Tg*ROP18 ((**D**–**F**) respectively).

A molecular docking study was carried out to investigate the possible mode of action of the tested drugs against *T. gondii*. The molecular docking study suggested that compounds **6** and **7** had greater affinity than the antiparasitic drug Megazol. Higher affinity results were noticed for *Tg*CDPK1 protein than for *Tg*ROP18 (Table 3 and Figure 10). The higher affinity of compound **7** against *Tg*CDPK1 and *Tg*CDPK1 can be explained by the formed Pi-Alkyl bond with HIS108 (in *Tg*CDPK1) and VAL266, LEU416 and ALA359 (in *Tg*CDPK1). *Tg*CDPK1 is a promising molecular target against *T. gondii* with known thiazoles co-crystalized inhibitors (PDB codes 5T6A, 5T6I and 5T6K). *Tg*CDPK1 is an excellent choice as a drug target because it differs from the human kinase proteins in the ATP binding site and the low bulky gatekeeper residue. Thus, it is possible to correlate the high affinity of compound **7** with the observed anti-*Toxoplasma* activity. The potential interaction between *Tg*CDPK1 protein and newly synthesized compounds could pave the way for associating these compounds’ in silico effects with the corresponding antiparasitic effect [29]. The electron-donating or electron-withdrawing substituents on the phenyl moieties as well as the steric bulkiness due to the presence of aromatic rings may play a role in the differential activity towards the parasite, as well as towards the host cells. While electronic effects could be considered to play a role in the action of the tested compounds, the electron-donating or electron-withdrawing capacity of the aromatic substituents did not appear to dramatically alter anti-*T. gondii* activity [30].

## 3. Materials and Methods

### 3.1. Drugs Synthesis and Analysis

#### 3.1.1. General

Standard procedures were used to purify commercially available solvents and reagents. All melting points were determined using an uncorrected Melt-Temp apparatus. Thin layer chromatography (TLC) was performed on aluminium plates with Silica Gel 60 F254 (Darmstadt, Germany, E-Merk, layer thickness of 0.2 mm) with UV light absorption detection. The IR spectra of the chemicals in a KBr matrix were recorded using an FTIR-8400s-Fourier transform infrared spectrophotometer-Shimadzu (Wichita, KS, USA). The Bruker (Billerica, MA, USA) spectrophotometer (400 and 600 MHz) was used to perform the NMR spectra, with TMS serving as an internal standard. A Finnigan LCQ spectrometer was used to collect the ESI mass spectrum data, while a Finnigan (Boston, MA, USA) MAT 95XL spectrometer was used to perform the EI mass spectra. The elementar Analysen-system GmbH-Vario EL III Element Analyzer (Jyväskylä, Finland) was used for the CHN analyses. All the chemicals used throughout the manuscript were purchased from Sigma-Aldrich (St. Louis, MO, USA)

#### 3.1.2. Synthesis and Characterization of Diethyl 4,4′-[(1,3,4-Thiadiazol-2,5diyl)bis(sulfanediyl)]dibutanoate (**2**)

Ethyl bromobutyrate (2.2 mmol) was added drop-wisely to the stirred solution of 2,5-dimercapto-1,3,4-thiadiazole (**1**) (1 mmol) in DMF (20 mL) and sodium hydride (2.2 mmol). The stirring continued for 2 h at room temperature, and then the mixture was refluxed for 8 h. The reaction was quenched by crushed ice and the resultant solid was filtered, washed with water, dried, and recrystallized from ethanol, affording the corresponding ester-based 1,3,4-thiadiazole **2** as colourless crystals. Yield 92%; m.p. 95–96 °C. IR (υ, cm^−1^): 2879, 2936 (C-H al), 1740 (C=O), 1634 (C=N), 1270 (C-O). ^1^H-NMR: δ_H_ 4.08–4.12 (q, 2H, *J* = 4 Hz, *J* = 8 Hz, OC**H**_2_), 3.32 (t, 2H, *J* = 4 Hz, SC**H**_2_), 2.06 (bs, 3H, CH_2_C**H**_3_), 1.87–1.93 (m, 2H, C**H**_2_CO), 1.78–1.82 (m, 2H, CH_2_C**H**_2_CH_2_) ppm. ^13^C-NMR: δ_C_ 170.99 (**C**=O), 164.86 (**C**=N), 63.63 (O**C**H_2_), 33.66 (S**C**H_2_), 27.61 (**C**H_2_CO), 25.88 (CH_2_**C**H_2_CH_2_), 20.94 (CH_2_**C**H_3_) ppm. EI MS (*m*/*z*): 378.49 (M+). Anal. Calcd for C_14_H_22_N_2_O_4_S_3_: C: 44.42; H: 5.86; N: 7.40. Found: C: 44.28; H: 5.99; N: 7.23.

#### 3.1.3. Synthesis and Characterization of 4,4′-[(1,3,4-Thiadiazol-2,5-diyl)bis(sulfanediyl)]dibutanehydrazide (**3**)

Hydrazine hydrate (50 mmol) was added to the solution of ester **2** (10 mmol) in ethanol (50 mL) under stirring. The reaction mixture was refluxed for 6 h. The solution was cooled, and the excess of ethanol was evaporated under reduced pressure. The resultant product was collected and recrystallized from ethanol lead acid hydrazide **3** as a white solid. Yield 90%; m.p. 189–191 °C. IR (υ, cm^−1^): 3250–3400 (NH, NH2), 1689 (C=O), 1627 (C=N). ^1^H-NMR: δ_H_ 9.02 (s, 1H, N**H**), 4.22 (bs, 2H, N**H**_2_), 3.27 (t, 2H, *J* = 8 Hz, SC**H**_2_), 2.17 (t, 2H, *J* = 8 Hz, C**H**_2_CO), 1.89–1.96 (quin, 2H, CH_2_C**H**_2_CH_2_) ppm. ^13^C-NMR: δ_C_ 166.82 (**C**=O), 147.80 (**C**=N), 29.16 (S**C**H_2_), 24.38 (**C**H_2_CO), 18.65 (CH_2_**C**H_2_CH_2_) ppm. EI MS (*m*/*z*): 350.44 (M^+^). Anal. Calcd. for C_10_H_18_N_6_O_2_S_3_: C: 34.27; H: 5.18; N: 23.98. Found: C: 34.59; H: 5.07; N: 24.22.

#### 3.1.4. General Procedure for the Synthesis of Thiosemicarbazide Derivatives **4** and **5**

Phenyl/methyl isothiocyanate (22 mmol) was added dropwise to a solution of the acid hydrazide **3** (10 mmol) in ethanol (50 mL), then the mixture was heated under reflux for 6–8 h. After cooling, the resultant precipitate was collected by filtration and purified by recrystallization from ethanol to yield the targeted acid thiosemicarbazide **4** and **5**.

*2,2′-(2,2′-(3,3′-(1,3,4-Thiadiazol-2,5-diyl)bis-(sulfanediyl)bis-(propane-3,1-diyl)bis-(hydrazine-2,1-diyl)bis-(N-phenyl-2-oxoethanethioamide)* (**4**). This compound was obtained as colorless crystals. Yield 89%; m.p. 247–248 °C. IR (υ, cm^−1^): 3209–3350 (NH), 1675 (C=O), 1648 (C=N), 1292 (C=S). ^1^H-NMR: δ_H_ 9.90 (s, 1H, N**H**), 9.62 (s, 1H, J = 4 Hz, N**H**), 9.57 (d, 1H, J = 4 Hz, N**H**), 7.12–7.47 (m, 5H, Ar-**H**), 3.21 (t, 2H, J = 8 Hz, SC**H**_2_), 2.32 (t, 2H, J = 8 Hz, C**H**_2_CO), 1.96–2.03 (quin, 2H, CH_2_C**H**_2_CH_2_) ppm. ^13^C-NMR: δ_C_ 173.66, 174.90 (**C**=O, **C**=S), 126.20, 126.45, 128.13, 128.24, 128.30, 128.49, 128.89, 129.12, 130.06, 130.68, 130.95, 134.13, 135.31, 137.31, 142.49 (Ar-**C**, **C**=N), 32.12 (S**C**H_2_), 31.95 (**C**H_2_CO), 24.64 (CH_2_**C**H_2_CH_2_) ppm. EI MS (m/z): 620.32 (M^+^). Anal. Calcd for C_24_H_28_N_8_O_2_S_5_: C: 46.43; H: 4.55; N: 18.05. Found: C: 46.71; H: 4.74; N: 17.98.

*2,2′-(2,2′-(3,3′-(1,3,4-Thiadiazol-2,5-diyl)bis-(sulfanediyl)bis-(propane-3,1-diyl)bis-(hydrazine-2,1-diyl)bis-(N-methyl-2-oxoethanethioamide)* (**5**). This compound was obtained as colorless crystals. Yield 86%; m.p. 192–193 °C. IR (υ, cm^−1^): 3200–3310 (NH), 1660 (C=O), 1645 (C=N), 1310 (C=S). ^1^H-NMR: δ_H_ 9.67 (bs, 1H, N**H**), 9.12 (d, 1H, J = 4 Hz, N**H**), 7.88 (d, 1H, J = 4 Hz, N**H**), 3.18 (t, 2H, J = 8 Hz, SC**H**_2_), 2.84 (d, 3H, J = 4 Hz, NC**H**_3_), 2.27 (t, 2H, J = 8 Hz, C**H**_2_CO), 1.92–1.99 (quin, 2H, CH_2_C**H**_2_CH_2_) ppm. ^13^C-NMR: δ_C_ 171.37, 173.44 (**C**=O, **C**=S), 137.30, 142.49 (**C**=N), 32.01 (S**C**H_2_), 31.91 (**C**H_2_CO), 30.81 (N**C**H_3_), 24.55 (CH_2_**C**H_2_CH_2_) ppm. EI MS (m/z): 496.45 (M^+^). Anal. Calcd for C_14_H_24_N_8_O_2_S_5_: C: 33.85; H: 4.87; N: 22.56. Found: C: 33.89; H: 4.75; N: 22.38.

#### 3.1.5. General Procedure for the Synthesis of Thiadiazole Derivatives **6**–**7**

A solution of cold concentrated sulfuric acid (15 mL) containing an appropriate thiosemicarbazide derivative **4**–**5** (1 mmol) was cooled at 0 °C and stirred for 30 min. Then, the mixture was allowed to reach room temperature for an additional 16 h, the reaction was quenched by ice-cold water, and the pH was adjusted to alkaline (pH =8) using an aqueous solution of ammonium hydroxide. The resultant precipitate was filtered; washed with water, and recrystallized from ethanol to yield the targeted *tris*-1,3,4-thiadiazoles **6** and **7**.

*2,5-Bis[(2-phenylamino-1,3,4-thiadiazol-5-yl)propylthio]-1,3,4-thiadiazole* (**6**). This compound was obtained as white solid. Yield 82%; m.p. 235–236 °C. IR (υ, cm^−1^): 3224–3292 (NH), 1624 (C=N). ^1^H-NMR: δ_H_ 10.13 (s, 1H, N**H**), 7.49–7.84 (m, 5H, Ar**H**), 3.30 (t, 2H, *J* = 6 Hz, SC**H**_2_), 3.02 (t, 2H, *J* = 6 Hz, C**H**_2_C=N), 2.15–2.19 (quin, 2H, CH_2_**CH**_2_CH_2_) ppm.

^13^C-NMR: δ_C_ 116.06, 126.44, 140.29, 141.04, 158.09, 163.97, 164.44 (Ar-**C**, **C**=N), 32.78 (S**C**H_2_), 28.15 (**C**H_2_C=N), 28.00 (CH_2_**C**H_2_CH_2_) ppm. EI MS (*m*/*z*): 584.83 (M^+^). Anal. Calcd for C_24_H_24_N_8_S_5_: C: 49.29; H: 4.14; N: 19.16. Found: C: 49.13; H: 4.06; N: 19.28 (Figures S1–S3).

*2,5-Bis[(2-methylamino-1,3,4-thiadiazol-5-yl)propylthio]-1,3,4-thiadiazole* (**7**). This compound was obtained as white solid. Yield 80%; m.p. 222–224 °C. IR (υ, cm^−1^): 3217–3290 (NH), 1636 (C=N). ^1^H-NMR: δ_H_ 9.87 (s, 1H, N**H**), 3.21 (t, 2H, *J* = 4 Hz, SC**H**_2_), 2.89–2.94 (m, 5H, C**H**_2_C=N, NC**H**_3_), 2.13–2.10 (quin, 2H, CH_2_**CH**_2_CH_2_) ppm. ^13^C-NMR: δ_C_ 155.67, 164.72, 168.31 (C=N), 38.69 (N**C**H_3_), 31.98 (S**C**H_2_), 28.31 (**C**H_2_C=N), 26.19 (CH_2_**C**H_2_CH_2_) ppm. ESI MS (*m/z*): 461.49 (M+H)^+^. Anal. Calcd for C_14_H_20_N_8_S_5_: C: 36.50; H: 4.38; N: 24.32. Found: C: 36.31; H: 4.50; N: 24.49 (Figures S4 and S5).

### 3.2. In Vivo Acute Toxicity Study

**Figure 11:**
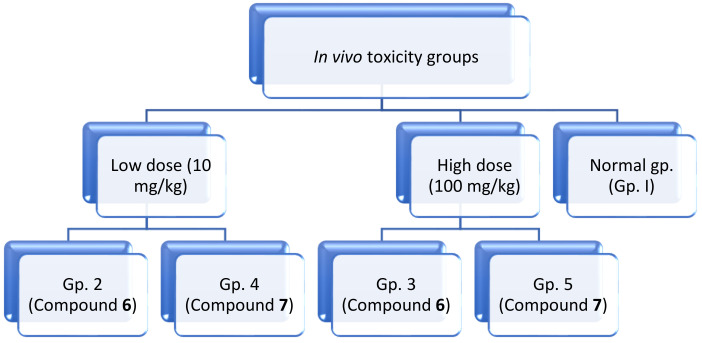
Design of the in vivo toxicity study.

Fifty male Swiss Albino mice with an average body weight of 20 ± 5 g (6 weeks old) were randomly chosen to assess and determine the appropriate dose of the tested drugs. This study was in accordance with the ARRIVE guidelines, the International Principles for Laboratory and Care of the European Community Directive (1986) and approved by the Animal Care and Use Committee (ACUC), Faculty of Science, Alexandria University (AU06210425902). Mice were divided into five groups (10 mice/group) according to the variations in the tested compounds and tested doses (Figure 11):

Group 1: Mice were given 100 μL normal saline (0.9%)
Group 2: Mice were given 100 μL compound 6 (10 mg/kg) body mass index (low dose).
Group 3: Mice were given 100 μL compound 6 (100 mg/kg) body mass index (high dose).
Group 4: Mice were given 100 μL compound 7 (10 mg/kg) body mass index (low dose).
Group 5: Mice were given 100 μL compound 7 (100 mg/kg) body mass index (high dose).

For five days, mice had oral administrations of the scheduled dosages every 24 h, whereas the control group received 0.9% sodium chloride doses. After five days, animals were sacrificed, and different organs (liver, brain and spleen) were excised to assess the histopathological changes [31].

### 3.3. Parasite

The *T. gondii* RH virulent strain was obtained from the Parasitology Laboratory, Theodor Bilharz Research Institute, Giza, Egypt. The strain was then maintained at Pharos University in Alexandria by the serial intraperitoneal passage of tachyzoites in Swiss Albino mice. The mice were sacrificed on the 4th day post-infection and their peritoneal fluid was washed three times with saline. A drop of the collected peritoneal exudate was added to the haemocytometer and the tachyzoites number was adjusted to be used for infecting the mice at a dose of 2500 tachyzoites/100 μL saline/mouse.

### 3.4. Drugs Preparation

The tested drugs, namely compounds **6** and **7**, were suspended in 100 μL of saline per mouse per dose and were orally administered by gavage needle, starting from the infection day and continuing for seven days.

### 3.5. Animal Grouping and Experimental Design

**Figure 12:**
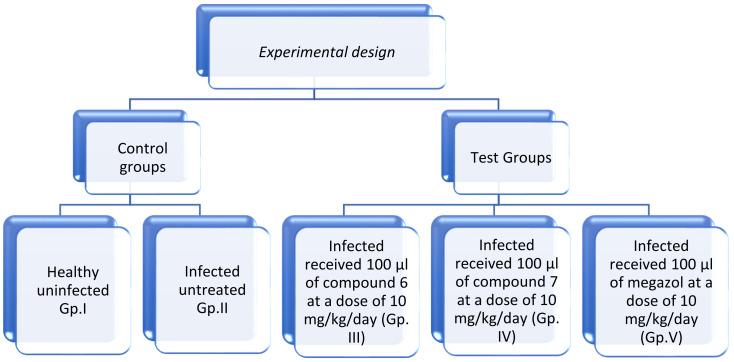
Experimental design of the animal groups.

Fifty laboratory-bred male Swiss Albino mice were enrolled in the study (6–8 weeks old and 20–25 g weight). The mice were distributed into four experimental groups (10 mice/group). Each mouse was infected intraperitoneally with the RH strain in a dose of 2500 tachyzoites/100 μL, except the uninfected control mice (healthy control). The mice groups were divided as follows (Figure 12):

- Group I: Healthy control, each mouse received 100 μL normal saline for seven days.
- Group II: Infected untreated control, each mouse received 100 μL normal saline (the vehicle of the used drugs) orally by gavage needle starting from the day of infection for seven days.
- Group III: Infected mice received 100 μL of compound 6 at a dose of 10 mg/kg/day orally by gavage needle starting from the day of infection for seven days.
- Group IV: Infected mice received 100 μL of compound 7 at a dose of 10 mg/kg/day orally by gavage needle starting from the day of infection for seven days.
- Group V: Infected mice received 100 μL of megazole at a dose of 10 mg/kg/day orally by gavage needle starting from the day of infection for seven days.

The mice were anaesthetized and sacrificed by cervical dislocation on the 8th day, i.e., 24 h after the last day of treatment.

Peritoneal exudates, liver, spleen, and brain were obtained from all the assigned groups for parasitological studies. However, the liver, spleen, and brain tissues were collected from all the groups and fixed in 10% formalin for the histopathological study. Peritoneal exudate from each mouse was fixed in glutaraldehyde for further morphological studies using a scanning electron microscope (SEM). On the other hand, the mice’s blood (5 mL) was collected through the retro-orbital plexus then centrifuged for 20 min at 4000× *g* to harvest the serum for inflammatory biomarker assessment.

Evaluation of treatment efficacy:

All the experimental groups were subjected to the following:

#### 3.5.1. Clinical Research

Variations in mice’s attitudes and food intake among studied groups were observed daily.

#### 3.5.2. Parasitological Study

##### Estimation of the Parasite Count

Tachyzoites were counted in Giemsa-stained impression smears of liver, spleen, and brain. The mean count was calculated in thirty oil immersion fields from each organ per each mouse (10 fields/slide and three slides/organ). The mean number was calculated for each subgroup [32].

##### Parasite Percent Reduction (%R)

The reductions percentages in the parasite count in peritoneal fluid, liver, spleen, or brain were calculated according to the following equation:

$$\%R=\frac{C-E}{C}\times 100$$

where *%R*: reductions percentage, *C*: parasites count in control group (untreated group) and *E*: parasites count in experimental groups of mice [33].

##### Morphological Study of *T. gondii* Tachyzoites

The peritoneal fluid of all infected subgroups was collected on the sacrifice day and examined by a light microscope at 400× to investigate the effect of the different treatments on the movement of *T. gondii* tachyzoites. The peritoneal fluid was then fixed in glutaraldehyde and prepared for examining the ultra-structure of the parasites by SEM [10].

#### 3.5.3. Inflammatory Biomarkers

The cytokine levels of TNF-α, IL-10, IL-6 and IL-1B in *T. gondii*-infected mouse sera were examined by enzyme-linked immunosorbent assay (ELISA) kits (R&D Systems, Minneapolis, USA) according to the manufacturer’s protocols. The reaction was measured by a microplate reader (Absorbance 96, Byonoy, Germany) at 450 nm [7,34].

#### 3.5.4. Histopathological Study

Specimens from different tissues (brain, liver, and spleen) were fixed in formalin (10%), dehydrated in ascending grades of ethyl alcohol, cleared in xylol, and then embedded in paraffin wax. Sections of 5 µm thickness were cut and stained using Ehrlich’s hematoxylin and eosin (H&E) stain [10].

### 3.6. Mode of Action of the Tested Compounds

#### Molecular Docking

A molecular docking study was used to assess the possible mode of action of the tested compounds against two main molecular targets of *T. gondii* which were the ROP18 kinase (*Tg*ROP18) with PDB code 4JBV and the Calcium-Dependent Protein Kinase 1 (*Tg*CDPK1) with PDB code 4JRN. These enzymes have significant effects on parasitic invasion and growth, which makes them an ideal target of the thiadiazoles compounds. Avogadro software was used for drawing and optimizing the three-dimensional structures of the tested compounds, while Autodock Tools v1.5.6 software (autodock.scripps, San Diego, California 92037, USA, 2018) was used to prepare the enzymes (receptors) and the tested compounds (ligands) by adding polar hydrogens and charges. Autodock Vina software was used to calculate the molecular docking where all the grid boxes had a 25 Å × 25 Å × 25 Å dimensions and were centered on the co-crystallized ligands. A value of 15 for the exhaustiveness parameter was used. To set the grid boxes, re-docking of the co-crystallized ligands in each receptor was done. All the calculations were performed in triplicates and Megazol was used as a reference drug (thiadiazole antiparasitic drug) [29].

### 3.7. Statistical Analysis

Data were presented as mean ± SD. Data analysis was done using SPSS version 20 (SPSS.exe, San Diego, CA 92037, USA, 2020). An ANOVA F-test was used to calculate the difference between quantitative variables among groups.

## 4. Conclusions

Multi-step synthesized *tris*-thiadiazoles **6** and **7** were investigated as antiparasitic drugs. Different biological experiments have been conducted to reveal that compound **7** has magnificent activity. It was proven that compound **7** reduced the parasite count by 82.6, 65.3, and 64.81% in the brain, liver, and spleen tissues, respectively. This activity could be explained by the high affinity of compound **7** against calcium-dependent protein kinase 1 (*Tg*CDPK1) and ROP18 kinase (*Tg*ROP18) (two main enzymes responsible for parasite invasion and egression). Although the initial concern and the restricted data were previously reported in vitro and have been focused on thiadiazole compounds as anti-*Toxoplasma* agents, the present work is the first in vivo study to confirm their effectiveness against *toxoplasma* infection in infected mice.

## Figures and Tables

**Table 1:** The parasite count and the percentage reduction in the organs of RH-infected mice.

	Liver (*n* = 10)	Spleen (*n* = 10)	Brain (*n* = 10)
Group II Mean ± SD	10.10 ± 1.20	5.40 ± 0.84	2.30 ± 0.67
Group III (Cpd 6) Mean ± SD R1%	6.60 ± 1.17 ^a,c,d^ 34.56	3.40 ± 0.96 ^a,c,d^ 37.03	1.10 ± 0.57 ^a,d^ 52.2
Group IV (Cpd 7) Mean ± SD R2%	3.50 ± 1.18 ^a,b,d^ 65.35	1.90 ± 0.57 ^a,b^ 64.81	0.40 ± 0.52 ^a^ 82.6
Group V Mean ± SD R3%	5.40 ± 0.84 ^a,b,c^ 46.53	2.60 ± 0.70 ^a,b^ 51.9	0.80 ± 0.63 ^a,b^ 65.3
F *p*	74.63 <0.001	27.51 <0.001	18.63 <0.001

GII: RH-infected control subgroup, GIII: infected group received drug **6**, GIV: infected group received drug **7**, % R1 percentage of reduction in infected group taken drug **6**, % R2 percentage of reduction in infected group taken drug **7**. n: initial number of mice in the subgroup, SD: standard deviation, F: F test (ANOVA) and *p* ≤ 0.05 (statistically significant), ^a^ Significant with subgroup II, ^b^ Significant with subgroup III, ^c^ Significant with subgroup IV, ^d^ Significant with subgroup V.

**Table 2:** Inflammatory response of the infected and non-infected mice groups.

Mice Groups	TNF-ɑ (ng/mL)	IL-10 (ng/mL)	IL-6 (pg/mL)	IL-1B (ng/mL)
Group I	1.87	1.37	393.87	1.20
Group II	2.81	2.41	626.81	11.14
Group III	2.55	2.05	579.26	10.63
Group IV	2.01	1.19	413.24	1.81
Group V	2.47	2.13	592.46	11.08

**Table 3:** Compounds affinity to *Tg*ROP18 and *Tg*CDPK1.

Compounds	Affinity *Tg*CDPK1 (kcal/mol)	Binding Features	Affinity *Tg*ROP18 (kcal/mol)	Binding Features
Megazol	−5.4	ASN190, ARG143 (Hydrogen bond)	−5.0	ALA359 (Hydrogen bond)
Compound **6**	−3.7	GLY213, GLU178 (Hydrogen bond)	−4.6	GLU300 (Hydrogen bond)
Compound **7**	−7.5	HIS108 (Pi-Alkyl bond)	−5.5	VAL266, LEU416, ALA359 (Pi-Alkyl bond)

## Supplementary Materials

The following supporting information can be downloaded at: https://www.mdpi.com/article/10.3390/molecules27072246/s1, Figure S1. IR of 2,5-Bis[(2-phenylamino-1,3,4-thiadiazol-5-yl)propylthio]-1,3,4-thiadiazole (**6**); Figure S2. ^1^HNMR of 2,5-Bis[(2-phenylamino-1,3,4-thiadiazol-5-yl)propylthio]-1,3,4-thiadiazole (**6**); Figure S3. ^13^CNMR of 2,5-Bis[(2-phenylamino-1,3,4-thiadiazol-5-yl)propylthio]-1,3,4-thiadiazole (**6**); Figure S4. ^1^HNMR of 2,5-Bis[(2-methylamino-1,3,4-thiadiazol-5-yl)propylthio]-1,3,4-thiadiazole (**7**); Figure S5. ^13^CNMR of 2,5-Bis[(2-methylamino-1,3,4-thiadiazol-5-yl)propylthio]-1,3,4-thiadiazole (**7**).

## Sample Availability

Samples of the compounds **6** and **7** are available from the authors.
