# Supplementary material for: Exploring the Antiparasitic Activity of Tris-1,3,4-Thiadiazoles against Toxoplasma gondii-Infected Mice
Source: Molecules. 2022 Mar 30;27(7):2246. doi: 10.3390/molecules27072246 (PMC9000608; doi:10.3390/molecules27072246)
Supplement: Supplementary file 1 [file molecules-27-02246-s001.zip › molecules-1644659-supplementary.pdf]

Supporting information

Exploring the antiparasitic activity of *tris*-1,3,4-Thiadiazoles against *Toxoplasma gondii* infected mice

Tahani M. Almutairi[1], Nadjet Rezki[2], Mohamed Reda Aouad[2], Mohamed Hagar[3*], Basant A Bakr[4], Moaaz T Hamed[5], Maha Khairy Hassen[6], Bassma H Elwakil[6*], Esraa Abdelhamid Moneer[6]

[1] Department of Chemistry, College of Science, King Saud University, P.O. Box 2455, Riyadh 11451, Saudi Arabia
[2] Department of Chemistry, Faculty of Science, Taibah University, Al-Madinah Al-Munawarah 30002, Saudi Arabia
[3] Department of Chemistry, Faculty of Science, Alexandria University, Alexandria 21321, Egypt
[4] Department of Zoology, Faculty of Science, Alexandria University, Alexandria 21321, Egypt
[5] Department of Botany and Microbiology, Faculty of Science, Alexandria University, Alexandria 21321, Egypt
[6] Department of Medical laboratory technology, Faculty of Applied Health Sciences Technology, Pharos University in Alexandria, Alexandria, Egypt
* Correspondence: BHE: bassma.hassan@pua.edu.eg ; MH: mohamedhaggar@gmail.com

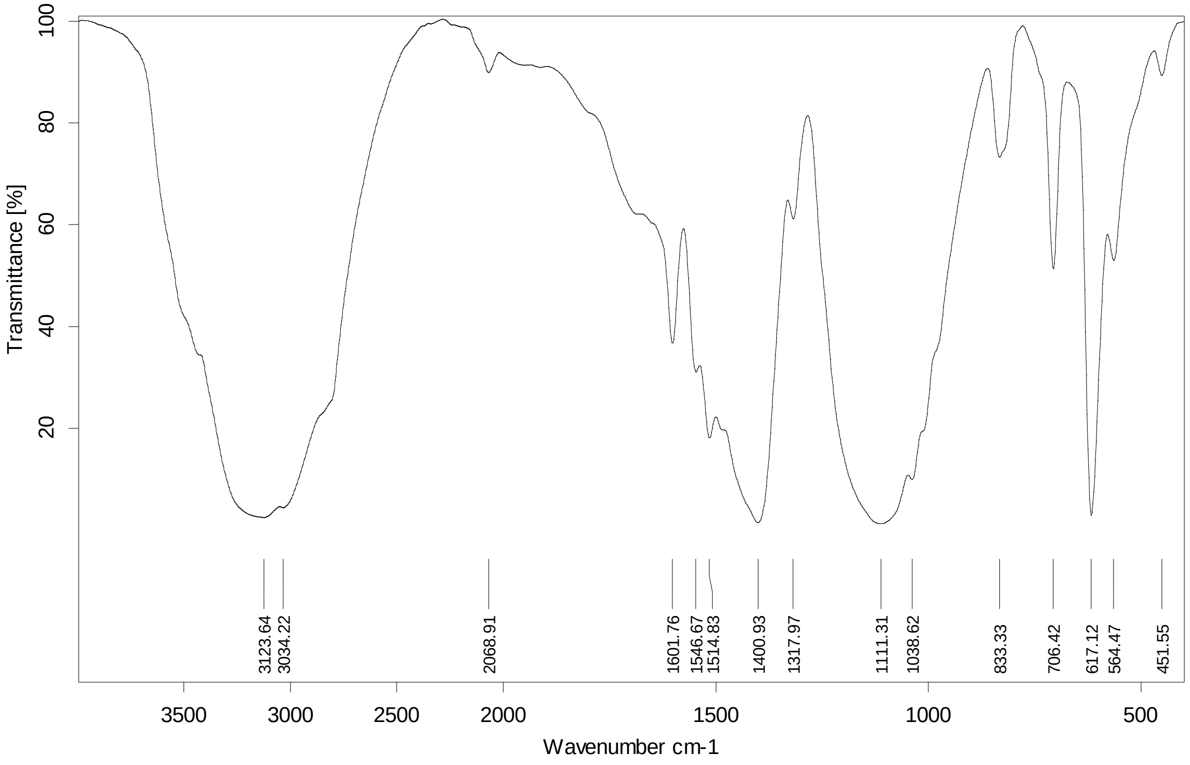

Figure S1. IR of 2,5-Bis[(2-phenylamino-1,3,4-thiadiazol-5-yl)propylthio]-1,3,4-thiadiazole (**6**).

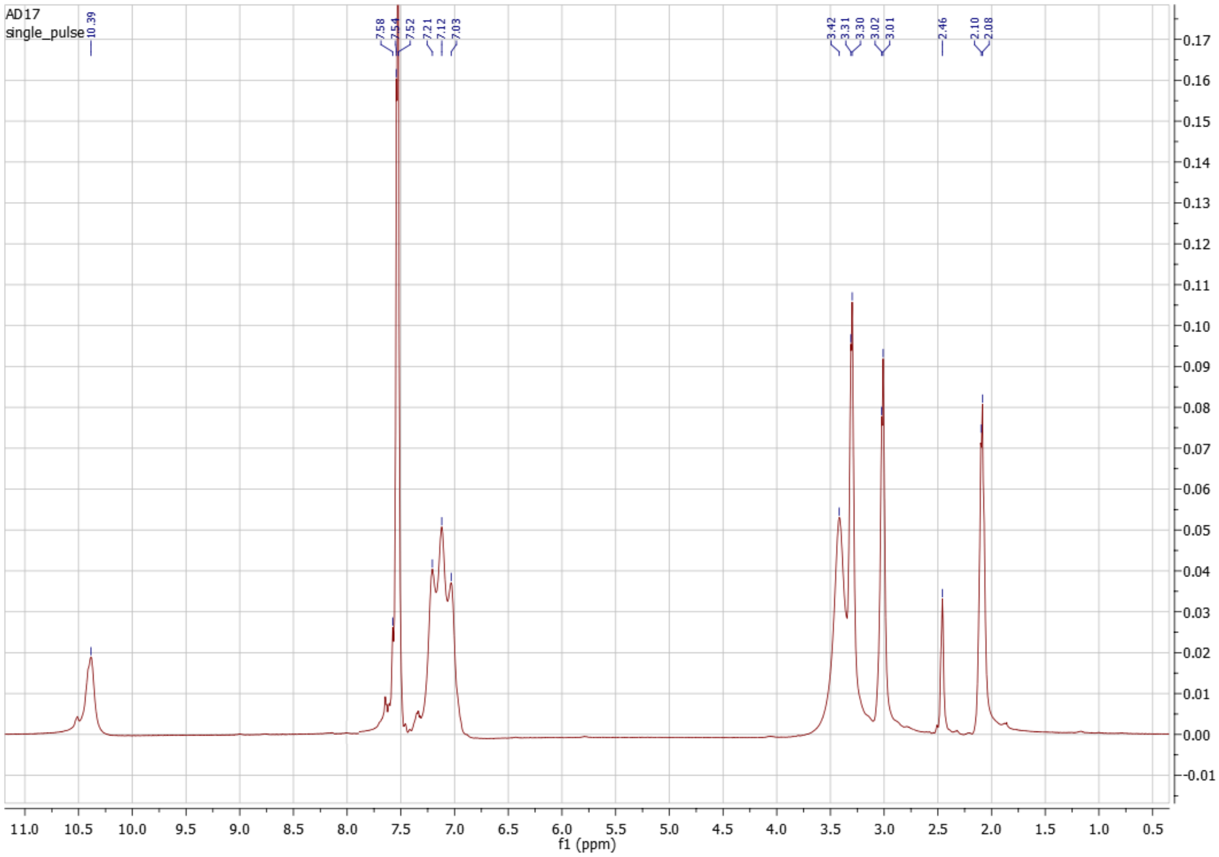

Figure S2. ^{1}HNMR of 2,5-Bis[(2-phenylamino-1,3,4-thiadiazol-5-yl)propylthio]-1,3,4-thiadiazole (**6**).

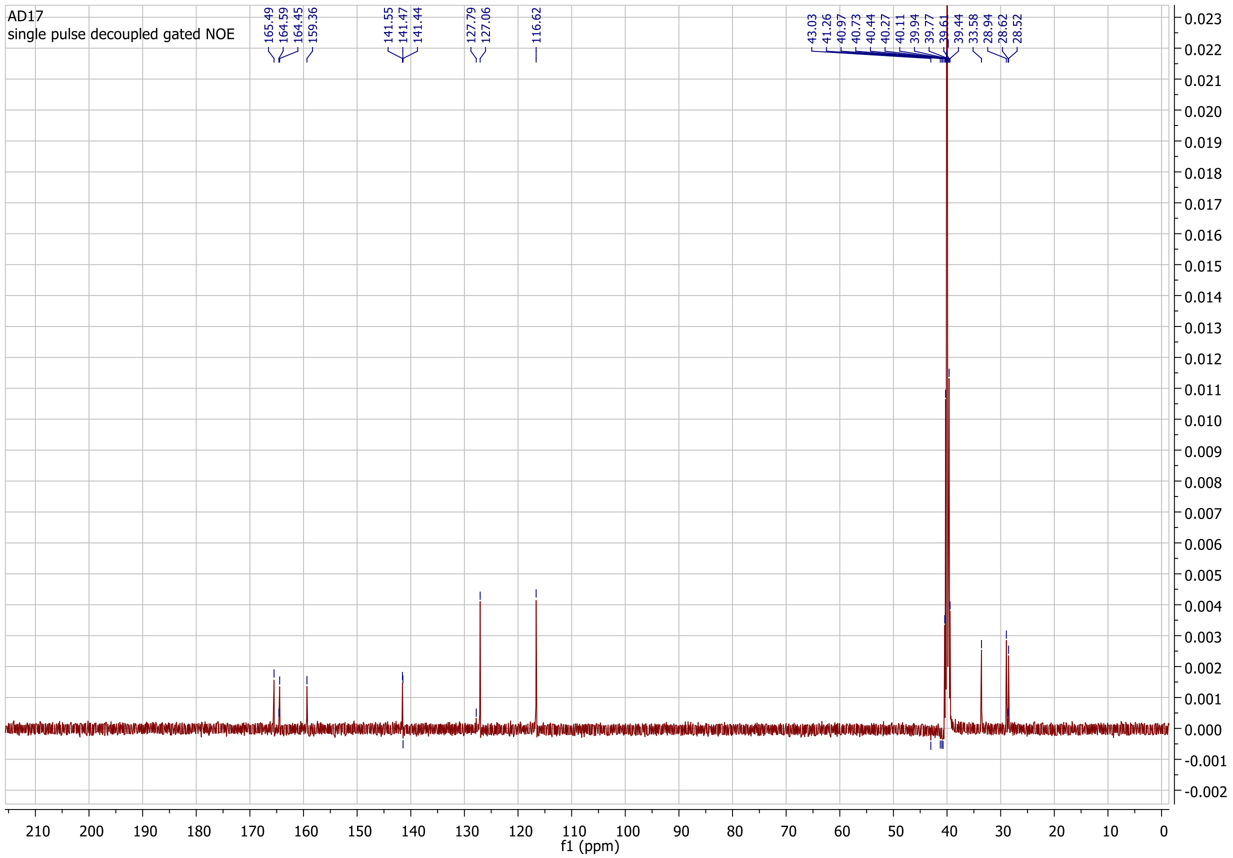

Figure S3. ^{13}CNMR of 2,5-Bis[(2-phenylamino-1,3,4-thiadiazol-5-yl)propylthio]-1,3,4-thiadiazole (**6**).

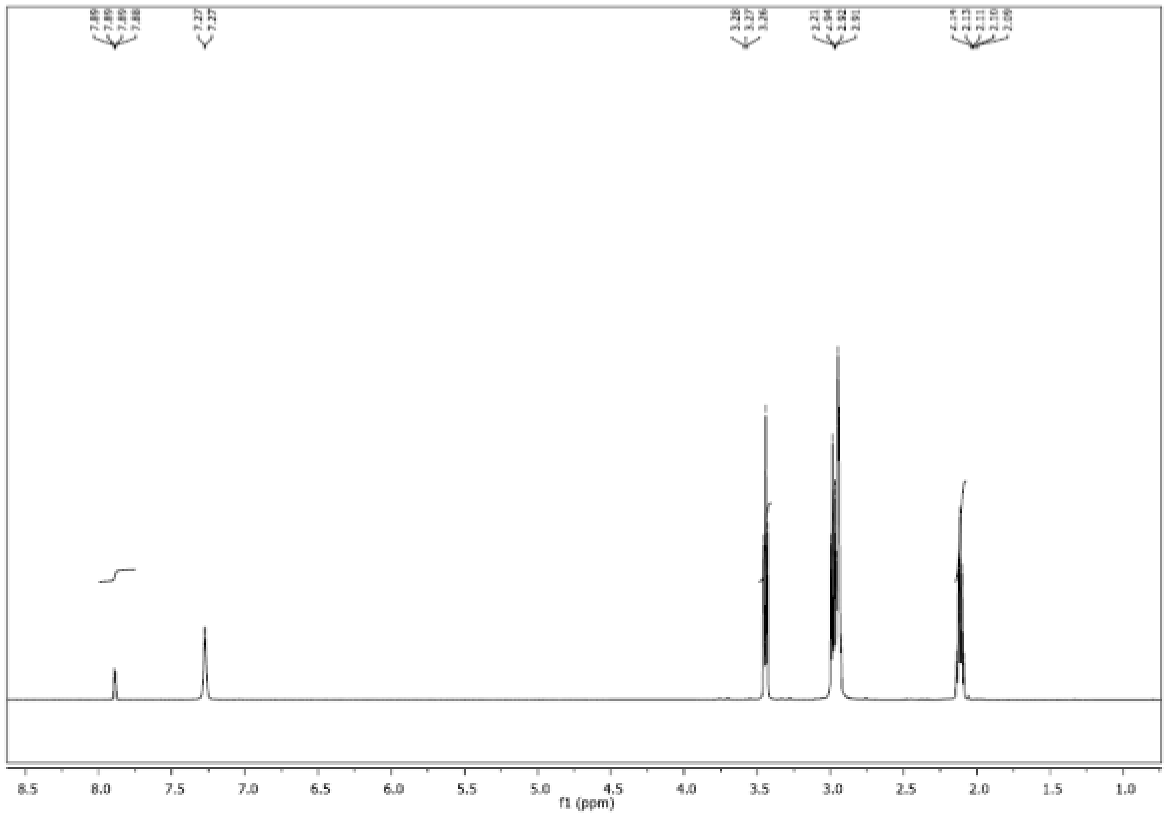

Figure S4. ^{1}HNMR of 2,5-Bis[(2-methylamino-1,3,4-thiadiazol-5-yl)propylthio]-1,3,4-thiadiazole (**7**).

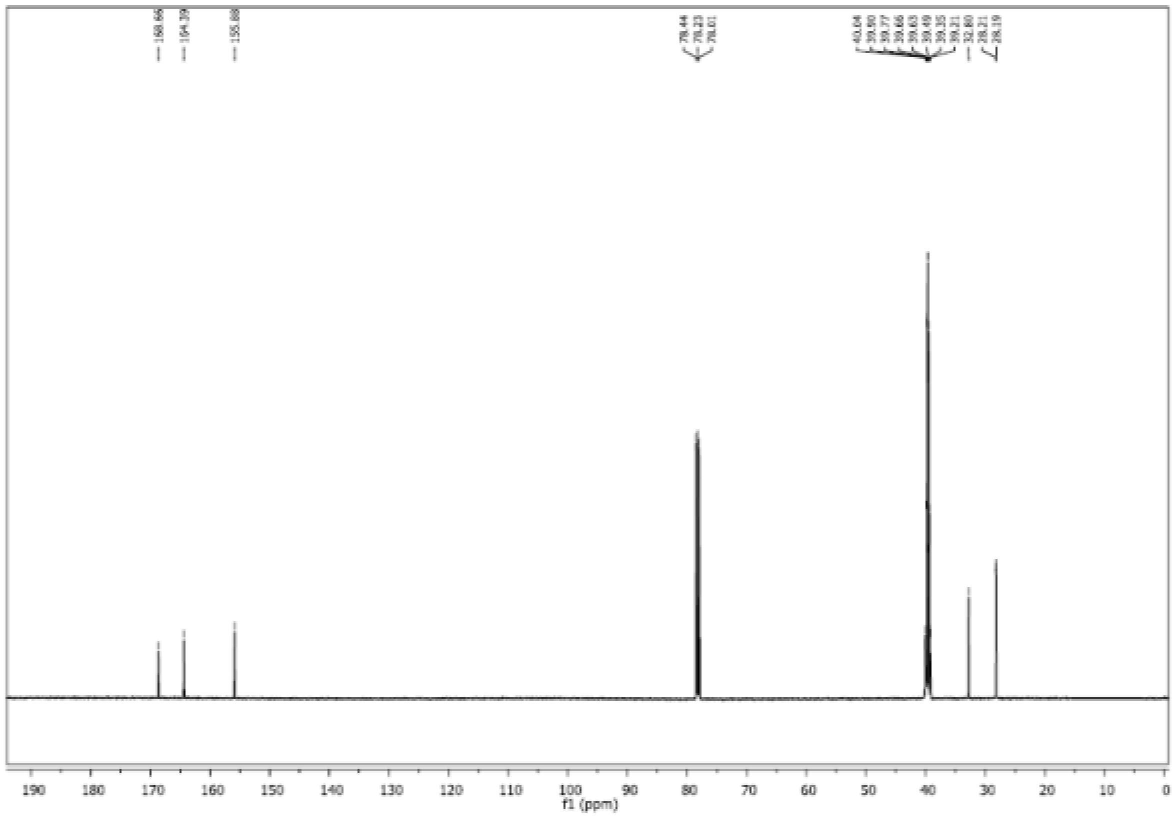

Figure S5. ^{13}CNMR of 2,5-Bis[(2-methylamino-1,3,4-thiadiazol-5-yl)propylthio]-1,3,4-thiadiazole (**7**).
